# Enabling culturally safe sexual health services in western Sydney: a protocol to improve STI treatment outcomes for Aboriginal young people

**DOI:** 10.1186/s40814-021-00847-7

**Published:** 2021-05-13

**Authors:** Ashley Ubrihien, Kylie Gwynne, David A. Lewis

**Affiliations:** 1 Faculty of Medicine and Health, University of Sydney, Sydney, NSW Australia; 2 Poche Centre for Indigenous Health, University of Sydney, Sydney, NSW Australia; 3 Faculty of Medicine and Health Sciences, Macquarie University, Macquarie Park, NSW Australia; 4 Western Sydney Sexual Health Centre, Western Sydney Local Health District, Parramatta, NSW Australia; 5 Westmead Clinical School, University of Sydney, Westmead, NSW Australia

**Keywords:** Sexual Health, Aboriginal, Barriers, Equity

## Abstract

**Background:**

Aboriginal people face challenges on several fronts when it comes to the health and wellbeing of their community, compared to the rest of the Australian population. This is no different in urban areas such as Australia’s largest urban Aboriginal community located in Blacktown, NSW, where sexually transmitted infections (STIs) remain an issue of concern. Across Australia, rates of infectious syphilis, human immunodeficiency virus (HIV), and hepatitis C infection have increased by 400, 260, and 15% respectively while gonorrhoea decreased 12% in the 5-year period from 2013 to 2017. This study explores how to address the barriers that prevent young Aboriginal people under 30 years of age from accessing STI treatment through Government Sexual Health Services.

**Methods:**

This qualitative study will use purposeful sampling to recruit 20 male and 20 female health consumers, 10 Aboriginal elders and 10 sexual health clinicians. This recruitment will be undertaken with the assistance of the local Government Health Services and local Aboriginal organisations. One-on-one semi-structured interviews will be undertaken by someone of the same gender in order to address cultural preferences. Data will be entered into NVivo and thematically analysed.

**Discussion:**

This study will seek to add to the literature that explores why young Aboriginal people do not access sexual health services. This study seeks to understand the experience of clinicians, Aboriginal elders and Aboriginal young people to provide practical policy and clinical redesign evidence that can be used to improve the experience and cultural safety of sexual health services in urban areas of Australia. The results of the qualitative research will be disseminated with the assistance of participating local Aboriginal organisations, and the findings will be published through peer-reviewed scientific journals and conference presentations.

**Trial registration:**

The study is approved by the Western Sydney Local Health District Human Research Ethics Committee (HREC/16/WMEAD/449) and the New South Wales Aboriginal Health and Medical Research Council’s Human Research Ethics Committee (1220/16).

## Background

In 2008, *The Closing the Gap report* delivered the Australian Parliament a stark picture of the health of Australia’s first people [1]. The scale of health inequity between Aboriginal people and the rest of the population manifested in a life expectancy gap of approximately 10 years [1]. Over the past 12 years since the release of that report, there has been little change in health outcomes for Aboriginal and Torres Strait Islander Australians (here after respectfully referred to as, Aboriginal) despite a whole of government focus on major goals articulated in the National Closing the Gap strategy [2]. In parallel, the rates of sexually transmissible infections (STIs) for Aboriginal people have continued to rise over this period [1]. Of particular concern are rates of infectious syphilis, human immunodeficiency virus (HIV) and hepatitis C infection which have increased by 400, 260 and 15% respectively while gonorrhoea has decreased 12% in the 5-year period from 2013 to 2017 [3]. The median age range at which each infection occurs in Aboriginal people is between 21 and 34 years of age [3]. There is a need to develop novel approaches to identify how services can better respond to the health care needs of this population.

Whilst the Aboriginal population across Australia represents 3% of the total population, incidence rates of STIs can range anywhere from 3 to 30 times higher than that of the non-Aboriginal population [3, 4]. According to the Australian Bureau of Statistics (ABS), the Western Sydney Local Government area (LGA) of Blacktown City Council has the largest urban population of Aboriginal people with approximately 9526 living in this area at the time of the 2016 Australian Census [5]. Although rates of STIs in urban Aboriginal communities are not as high as remote communities, which experience rates of infection that are 5 to 500 times higher than the general population, sexual health should be prioritised within Blacktown LGA due to the relatively high number of Aboriginal people living in this concentrated urban environment [4]. Another important factor worthy of consideration when assessing the scale of the public health problem is the likelihood that members of a community would be sexually active and therefore at risk of acquiring and transmitting STIs to other members of that community. The ABS identifies the median age of Aboriginal people living in this LGA as 19 years of age which means that there is significant number of people who may be at risk of STI acquisition [5]. The New South Wales (NSW) Government’s *NSW STI Strategy 2016–2020* also identifies Aboriginal people as a priority population in the strategy and in particular focuses upon young people [6].

Aboriginal people in urban communities are often not the focus of research because the funding priorities of the Commonwealth Government tend to direct significant funding towards remote communities and leave state and territory governments to address health needs in urban areas [4]. Aboriginal people in Western Sydney are highly urbanised yet still face significant barriers to healthcare. They are younger than the wider population, experience high rates of unemployment and generally have larger families [5].

One of the barriers to accessing sexual health services appears to be shame and stigma [7–10]. The themes of shame and stigma have been identified by Bell et al. in a study of 35 young Aboriginal people in the Northern Territory where the authors specifically explored the reasons why young people did or did not engage in STI testing in this region. In a sample of 45 young Aboriginal people, surveyed by Mooney-Somers et al., one third of the participants indicated that shame would be associated with having an STI and having to seek treatment [7]. This is also reflected in the mixed method study of Su et al. who found that the shame of many Aboriginal men was associated with the possibility of being seen at sexual health clinics and inferences of weakness and disease that might be associated with such a visit [9].

There is a significant gap in the published literature in terms of studies that seek to understand the issues that prevent Aboriginal people participating in sexual health care through the collection of directly obtained evidence. Many of the studies reviewed, including those undertaken by Bailie et al., Nattabi et al., Buhrer-Skinner et al. and Richmond et al., rely on the response of clinicians in quality improvement processes to guide the conclusions of their research [11–14]. These studies focus on the process aspects of clinical service delivery and how quality improvement principles can be followed to improve quality; however, these tools provide limited insight into the barriers that prevent young Aboriginal people from attending the services [11–14]. The conclusions of research using this approach may have some merits; however, they are likely to miss the important nuances that could be obtained by engaging health consumers in the research process. It is possible to engage health consumers in quality improvement processes, and this would give those undertaking such a study an objective source of evidence.

The status of sexual health amongst young Aboriginal people in Western Sydney is an area that has not been explored in any great depth within the published literature. The study undertaken by Biggs et al. provides a cross-sectional view of Aboriginal people attending public sexual health services in Western Sydney through an incentive-based program called ‘The Deadly Liver Mob’ [15]. The Deadly Liver Mob program primarily focused on education concerning hepatitis C infection for people who are currently or have been injecting drug users [15]. For these reasons, the group screened for STIs in this study are a distinct sub-population of the Aboriginal Community, making it hard to generalise the results of this study [15]. This study also relied on the retrospective review of routine clinical data, so there was no ability to explore questions that were not addressed in a limited data set.

This study will seek to add to the literature by exploring why young Aboriginal people do not access sexual health services. This study seeks to understand the experience of clinicians, Aboriginal elders and Aboriginal young people to provide practical policy and clinical redesign evidence that can be used to improve the experience and cultural safety of sexual health services in urban areas of Australia.

## Methods

This qualitative study will utilise content analysis design and undertake semi-structured one-on-one interviews with up to 40 health consumers (potential or current service users), 10 clinicians and 10 community elders or until a saturation point is reached. Inclusion criteria for health consumers will be that they need to be between 18 and 29 years of age at the time of enrolment into the study. The semi-structured interview guide was developed with feedback from Aboriginal staff and Clinical Staff with specialist knowledge of sexual health. Wherever possible, we will be using Aboriginal interviewers to create culturally safe spaces for study participants to respond to questions. All cohort groups will be interviewed using a semi-structured interviewer-assisted questionnaire with the aim of answering the following questions:

1. What prevents Aboriginal people in Western Sydney from accessing government sexual health services?
2. What would enable Aboriginal people in Western Sydney to access mainstream services?
3. Would changes in service modality make services more accessible?
4. What do participants see as the most important things that could be implemented to improve sexual health services in Western Sydney?

A target number was purely set to ensure gender equity amongst study participants, and data collection will cease once saturation has been reached. The study investigators have adequate qualifications and experience to undertake a qualitative study in the area of sexual health and have significant expertise in community engagement and co-design. The questionnaire is worded in plain English to ensure that potential participants with low literacy can also participate. Interviews will be audio recorded, and results of this study will be analysed using the software program, NVivo (QSR International, Melbourne, Australia), so that major themes can be identified and thematically analysed.

This data will create translational research that will assist service delivery models in publicly funded Sexual Health services in Western Sydney and perhaps more widely.

Study participants will be free to withdraw from the study at any point up to where audio files are destroyed, post the transcription process. Participants will not be able to withdraw after this point because individual transcripts will only be coded with the participant’s interview cohort, gender and a sequential number; therefore, any individual submission will not be able to be identified for withdrawal.

This study will be conducted in a manner that safeguards the anonymity of participants by ensuring that interview responses do not contain any identifying marks apart from an indication of age and sex. This will assist in protecting the privacy of Aboriginal people who choose to participate in the study. The value of Aboriginal interpretation is integral to this project’s success, and this will be achieved through the engagement of Aboriginal research assistants, consultation about the study design and through the engagement of Aboriginal people in the analysis of data and the final conclusions of the study. Involvement in the governance of the study is also of paramount importance, and every effort will be made to ensure participation.

The investigators anticipate that some risks may be posed by participants disclosing sensitive information particularly around the conduct of illegal activities, a history of sexual or physical abuse or need to have sexual health conditions treated. Should a disclosure of illegal activity be made, the interview would be immediately terminated in order to ensure that the participant did not further incriminate themselves. If the disclosure relates to any form of abuse that falls under the definitions of Section 27 of the *NSW Children and Young Persons (Care and Protection) Act 1998*, the matter will be immediately reported to the NSW Department of Families and Communities, and referrals would be offered to relevant support services [16]. In order to maintain the confidentiality of participants, all data collected will be de-identified and contain no potentially identifying information apart from age and sex. It is important to highlight to participants the risk that is posed by them disclosing illegal activities because of the need of study investigators to report such activities to the relevant government agency.

## Dissemination of results

The results of this study will be disseminated through several different media including meetings with study participants, local elders and Aboriginal groups, peer-reviewed publications, presentation of results at conferences, and through summaries presented via social media.

The contribution of local Aboriginal people will be acknowledged in all publications to reflect the unique contribution that they provide, and co-authorship of academic papers will be offered, consistent with University of Sydney policy on the matter. The research will be conducted in accordance with the National Health and Medical Research Council (NHMRC) guidelines for ethical research with Aboriginal people [17]. Plain English resources and information meetings will be undertaken with communities involved in this study to provide a summary of the results of the findings.

## Discussion

This study will provide benefits to Aboriginal people by enabling local health services to better understand and allocate resources to meet the needs of young Aboriginal people through the acquisition of more robust study data concerning how Sexual Health Services may best be delivered.

The results of this study will be significant in informing urban health services on the needs of young Aboriginal people in terms of how they prefer to access public sexual health services and the barriers that currently exist that may prevent them from using existing services. Much of the research based both in remote areas and urban areas relies on the experiences of clinicians rather than the lived experience of young Aboriginal people themselves and may miss important aspects of interpretation that only occur when Aboriginal people share their insights. These insights will be deepened in this study by involving local Aboriginal people in the interpretation of the de-identified results of the study.

This engagement project will provide a set of documents that will be informed by the local community and provide policy makers with practical measures that can be implemented to improve the cultural appropriateness and clinical care of young Aboriginal people. This study will also address important policy questions and directly inform on the delivery of healthcare to young Aboriginal people in urban settings.

## Data Availability

Not applicable. This manuscript does not contain any data.

## Abbreviations

ABS: Australian Bureau of Statistics
HIV: Human immunodeficiency virus
LGA: Local government area
NHMRC: National Health and Medical Research Council
NSW: New South Wales
STIs: Sexually transmitted infections
WSLHD: Western Sydney Local Health District
